# MAMDC2‐AS1 Induces Cuproptosis in Relapsed and Refractory Multiple Myeloma

**DOI:** 10.1002/cnr2.70216

**Published:** 2025-05-08

**Authors:** Yifei Chen, Jun Liu, Ying Zhu

**Affiliations:** ^1^ Department of Hematology Jiangdu People's Hospital Yangzhou City China

**Keywords:** cuproptosis, lncRNA, MAMDC2‐AS1, prognosis, RRMM

## Abstract

**Background:**

Multiple myeloma is a malignant disorder involving the uncontrolled proliferation of plasma cells in the bone marrow. Prognosis remains poor for individuals with relapsed and refractory multiple myeloma (RRMM), and the underlying mechanisms are yet to be fully understood.

**Methods:**

We collected bone marrow RNA‐Seq data from a total of 557 patients with MM from the GEO database (GSE24080) for further analysis, dividing them into relapsed/refractory and control groups. Additionally, we collected bone marrow samples from 57 MM patients to validate the performed RNA‐Seq data analysis.

**Results:**

RNA‐Seq analysis of patients with RRMM revealed a significant upregulation of genes associated with cuproptosis. Using the LASSO Cox regression method, several long noncoding RNAs (lncRNAs) were identified that influence copper‐induced cell death. Based on these lncRNAs, patients were stratified into high‐risk and low‐risk groups. The high‐risk group exhibited a significantly worse overall survival (OS) compared to the low‐risk group, with a *p*‐value of less than 0.001. Our statistical analysis, incorporating LASSO Cox regression, indicated that among these lncRNAs, MAMDC2‐AS1 was particularly noteworthy due to its strong correlation with OS (*p*‐value < 0.01). Further validation using qPCR and survival analysis established MAMDC2‐AS1 as a strong predictor of prognosis in MM. This finding suggests that MAMDC2‐AS1 can serve as a potential independent biomarker for RRMM. The qPCR data validated the RNA‐Seq findings and uncovered the significance of MAMDC2‐AS1 in the prognosis of this disease.

**Conclusion:**

MAMDC2‐AS1 plays a significant role in RRMM. Promisingly, Bortezomib, Bosutinib, Crizotinib, and DMOG have demonstrated promising efficacy in addressing advanced cases.

## 1 Introduction

Multiple myeloma (MM) is a tumor originating from plasma cells, a type of white blood cell specialized in antibody production [1, 2]. Typically, the median age at diagnosis is around 65–70 years; although MM can also present in younger individuals and this condition often results in skeletal damage, functional impairment, and symptoms such as anemia, kidney dysfunction, and a weakened immune system [2, 3]. In clinical practice, MM is commonly staged using the International Staging System (ISS), which categorizes patients into three groups (stage I, II, or III) based on serum albumin and β2‐microglobulin levels [2]. Due to advancements in therapies like proteasome inhibitors, immunomodulatory agents, and monoclonal antibodies, survival outcomes in MM have significantly improved, with many patients now achieving median OS times of approximately 5–7 years [4, 5]. Nevertheless, disease heterogeneity remains a critical factor, and patients with high‐risk features or advanced stages may still face significantly shorter lifespans, underscoring the need for continuous research and therapeutic innovation in this challenging malignancy.

While chemotherapy, radiation therapy, and stem cell therapy can be commonly used to overcome MM, the recurrence of the disease in a relapsed and refractory form poses a major challenge [2]. Relapsed multiple myeloma refers to the return of the disease after an initial response to treatment, while refractory multiple myeloma describes a situation where the disease fails to respond adequately to therapy or relapses within a short period [6]. Patients with RRMM generally face a poorer prognosis and significantly reduced OS [7]. The mechanisms that contribute to RRMM are not yet fully understood, but evidence suggests they include genetic mutations in tumor cells, immune system evasion, and abnormalities within the bone marrow microenvironment, all of which can render standard treatments less effective [8, 9]. Additionally, drug resistance frequently occurs in RRMM, further complicating treatment by reducing the effectiveness of conventional therapies [8]. Consequently, patients with RRMM often have severely limited therapeutic avenues, underscoring the urgent need for new and more effective treatment strategies to overcome resistance and improve prognosis [10, 11].

Despite significant advances in immunotherapies and targeted treatments—such as novel drug combinations, monoclonal antibodies, chimeric antigen receptor T (CAR‐T) cells, and precision medicine approaches—RRMM remains a formidable clinical challenge [10, 11]. Although these therapies have shown promise in extending survival, overcoming drug resistance, and improving patient outcomes, there is still a critical need to elucidate the mechanisms that underlie RRMM progression [9]. Recent evidence suggests that dysregulated long noncoding RNAs (lncRNAs), particularly those involved in cuproptosis—a newly characterized form of cell death—may critically influence MM pathogenesis and therapy resistance [12]. Consequently, understanding the specific role of cuproptosis‐related lncRNAs in RRMM could provide novel insights into disease progression and identify new targets for more effective therapeutic interventions, ensuring that further research aligns closely with this emerging focus on cuproptosis and lncRNA regulation in advanced MM [6].

Cuproptosis, a recently characterized type of cell death [13], has been implicated in various cancers such as liver, lung, and breast cancer, opening new possibilities for therapeutic targeting [14]. lncRNAs play important roles in cancer, but research on cuproptosis‐related lncRNAs (CRLPMs) in RRMM remains limited [14]. Despite their critical roles in cellular processes, research on CRLPMs and their prognostic significance in RRMM is still in its early stages. These lncRNAs have the potential to serve as important biomarkers and therapeutic targets, but their involvement in the disease's progression and patient outcomes remains underexplored. Further investigation is necessary to understand the full impact of CRLPMs on MM and to develop more effective treatment strategies.

In this project, we performed single‐center research, gathering bone marrow RNA‐Seq data from 557 MM patients in the GEO database (GSE24080) for cuproptosis‐related analysis. Moreover, we collected bone marrow samples from 57 MM patients at our medical center for validation, focusing on immunotherapy responses and drug sensitivity. Through these investigations, our research aims to uncover potential drug targets for RRMM.

## 2 Materials and Methods

### 2.1 Patients

In this retrospective real‐world study, we analyzed data from 57 MM patients at our center and 557 MM patients from the GEO database, covering the period from September 3, 2021, to January 27, 2023. We collected bone marrow samples from two distinct groups: one group of RRMM patients and another group of newly diagnosed or non‐RRMM patients. Diagnoses were confirmed through bone marrow morphological analysis, and data collected included patient demographics, MM subtype, ISS stage, and survival outcomes (Table 1). Median values were used for classification. Informed consent was obtained, and the study adhered to the ethical guidelines of Jiangdu People's Hospital (Approval No. 200812931) and the Declaration of Helsinki.

**TABLE 1:** Baseline characteristics and procedure characteristics.

Variables	Total (*n* = 57)
Age, *n* (%)	
< 65	30 (60)
≥ 65	27 (40)
Gender, *n* (%)	
Female	36 (46)
Male	21 (54)
BMI (mean ± SD)	23.12 ± 1.3
Classification	
IgA	20 (30)
IgM	15 (34)
IgG	15 (31)
L‐κ	7 (31)
ISS stage, *n* (%)	
I	12 (30)
II	20 (34)
III	25 (31)
DS stage, *n* (%)	
IA	4 (30)
IIA	25 (34)
IIIA	21 (31)
IIIB	7 (5)

Abbreviations: BMI, Body Mass Index; DS, Disease‐Severity; ISS, International Staging System.

After confirming the diagnosis of MM and before starting chemotherapy, we collected BM from the patients once more for this study. The inclusion criteria for the 57 MM patients in this retrospective real‐world study were as follows:

### 2.2 Inclusion Criteria

1. Diagnosis: Patients must have been diagnosed with MM confirmed through BM morphological analysis.
2. Study Period: Patients included in the study received treatment from September 3, 2021, to January 27, 2023.
3. Data Availability: Complete datasets, including variables such as age, gender, body‐mass index (BMI), MM subtype, ISS stage, and survival outcomes, must be available.
4. Ethical Consent: Informed consent must have been secured from all patients or their immediate family members, if the patient was unable to give consent themselves.

### 2.3 Exclusion Criteria

1. Previous Cancer Diagnosis: Patients with a history of other malignancies were excluded to avoid confounding effects on the results of the current study.
2. Incomplete Medical Records: Patients with incomplete medical records or missing critical data (e.g., age, gender, treatment history) were excluded.
3. Concurrent Severe Medical Conditions: Patients with severe concurrent medical conditions (e.g., severe cardiovascular disease, uncontrolled diabetes mellitus) that could significantly impact OS and response to treatment were excluded.
4. Non‐Compliance with Treatment: Patients who were non‐compliant with prescribed treatments or follow‐up schedules were excluded to maintain the consistency of treatment effects in the study.
5. Recent Participation in Clinical Trials: Patients who had recently participated in other clinical trials were excluded to prevent interference from experimental treatments that could affect the study outcomes.

### 2.4 Sample Collection

BM samples from MM patients were collected at the time of initial diagnosis. After the samples were centrifuged at 1500 rpm for 5 min, the supernatant was carefully removed for further processing. The BM cells then underwent erythrocyte depletion by incubating with erythrocyte lysis buffer (Solarbio, China) for 5 min. These steps ensured the proper isolation of bone marrow mononuclear cells (BMMCs) and the extraction of high‐quality RNA for further analysis.

### 2.5 RNA Isolation and qRT‐PCR

BMMCs were first subjected to total RNA extraction using TRIzol reagent (Invitrogen, USA), following the manufacturer's guidelines. Reverse transcription was then conducted with the 5× All‐In‐One RT MasterMix (abm, Canada), including a temperature step to deactivate the RT enzyme. Quantitative PCR (qPCR) was performed using 2× SYBR Green qPCR Master Mix (Low ROX) (Bimake, China), with carefully controlled thermocycling conditions and gene‐specific forward/reverse primers. Relative gene expression was determined using the 2^−ΔΔCt^ method, normalized to GAPDH as the reference gene [15]. Primer sequences for each target gene are provided in Table S1.

### 2.6 Identification and Evaluation of Cuproptosis‐Associated lncRNAs

We employed the “limma” package in R to detect CRLPMs by calculating Pearson correlations (|cor| > 0.4, *p* < 0.001) between lncRNAs and cuproptosis‐related genes. Additionally, Sankey plots were generated with the “ggplot2,” “ggalluvial,” and “dplyr” packages to provide a clear visual representation of these gene–lncRNA relationships.

### 2.7 Cox Model Construction

To ensure an unbiased analysis, samples were randomly allocated into training and validation cohorts using the “caret” package in R. Univariate Cox proportional hazards regression (with the “survival” package) was then applied to assess survival outcomes for each CRLPM. To minimize overfitting, we utilized the “glmnet” package to perform LASSO‐penalized Cox regression, identifying the optimal penalty (λ) through 10‐fold cross‐validation. Subsequently, multiple stepwise Cox regression analyses were carried out to generate the CRLPM library, and a risk scoring model was formulated based on the LASSO risk score. Each patient was then assigned a risk score according to this model.

### 2.8 Validation of RNA‐Seq Data With Local Cohort

The training and validation cohorts were classified into high‐ or low‐risk groups based on the median risk score derived from the training dataset's coefficients. Prognostic performance for the CRLPMs was evaluated in both cohorts using Kaplan–Meier (KM) survival analysis. Receiver operating characteristic (ROC) curves were generated to gauge the model's accuracy, with area under the curve (AUC) values calculated via the “survival ROC” and “time ROC” packages in R. Principal component analysis (PCA), visualized with the “scatterplot3D” package, confirmed the validity of the risk models. Progression‐free survival (PFS) was assessed, and the C‐index was used to verify the model's reliability in both the validation cohort and the entire study population.

### 2.9 Nomogram Construction to Predict Risk of MM

The independent prognostic utility of the risk model was examined through univariate and multivariate Cox regression analyses. Based on these findings, a nomogram was constructed using the “rms,” “regplot,” and “survival” packages in R, incorporating the results from both regression approaches. This nomogram serves as a visual predictive tool, allowing clinicians to estimate patient outcomes based on individual risk factors, thus enhancing personalized treatment strategies for patients with MM.

### 2.10 Association Between Prognostic Risk Score and Clinical Status

To determine the model's applicability across various clinical stages in MM patients, we investigated how clinical stage correlates with the prognostic risk score. Both univariate and multivariate Cox regression analyses were conducted to evaluate the impact of the risk score and clinical stage on patient outcomes. This approach ensured that the risk score's prognostic utility was assessed while accounting for clinical stage as a covariate, thereby confirming the model's relevance across diverse patient populations.

### 2.11 Pathway and Functional Analysis

Using the “limma” R package, we identified differentially expressed genes (DEGs) between the high‐ and low‐risk cohorts, applying the criteria of |log2(fold change)| > 1 and a false discovery rate < 0.05. Subsequent functional enrichment analysis was conducted with the “clusterProfiler,” “org.Hs.eg.db,” and “enrichplot” packages, focusing on Gene Ontology (GO) [16] and Kyoto Encyclopedia of Genes and Genomes (KEGG) pathway analysis [17]. This approach elucidated the molecular mechanisms underlying the identified DEGs.

### 2.12 Drug Sensitivity Assessment

To explore the clinical relevance of the CRLPMs in treating RRMM, we estimated the half‐maximal inhibitory concentration (IC50) for various agents using the “pRRophetic” R package and its dependencies (“car”, “ridge”, “preprocessCore”, “genefilter”, and “sva”). A total of 138 drugs—including midostaurin, temsirolimus, tipifarnib, and imatinib—were examined. Differences in IC50 values between groups were assessed with the Wilcoxon signed‐rank test, and results were visualized as boxplots via “ggplot2”. These findings provide insights into how the CRLPM‐based risk stratification may inform drug selection in RRMM therapy.

### 2.13 Statistical Analysis

Categorical data were reported as proportions and analyzed using the *χ* ^2^ test. Continuous variables were presented as either medians with mean ± standard deviation or quartile ranges, according to their distribution. For group comparisons, one‐way ANOVA was employed when data followed a normal distribution, whereas the Kruskal‐Wallis test was used for skewed data. Post hoc tests were then conducted to perform pairwise comparisons among the four stages. KM curves visualized cumulative mortality, with survival differences assessed by log‐rank tests. Univariate and multivariate Cox regression models adjusted for survival responses, estimating OS. Forest plots displayed prognostic covariate significance, and functional relationships were analyzed using constrained cubic spline alignments with the “rms” package and LASSO analysis.

**FIGURE 1:**
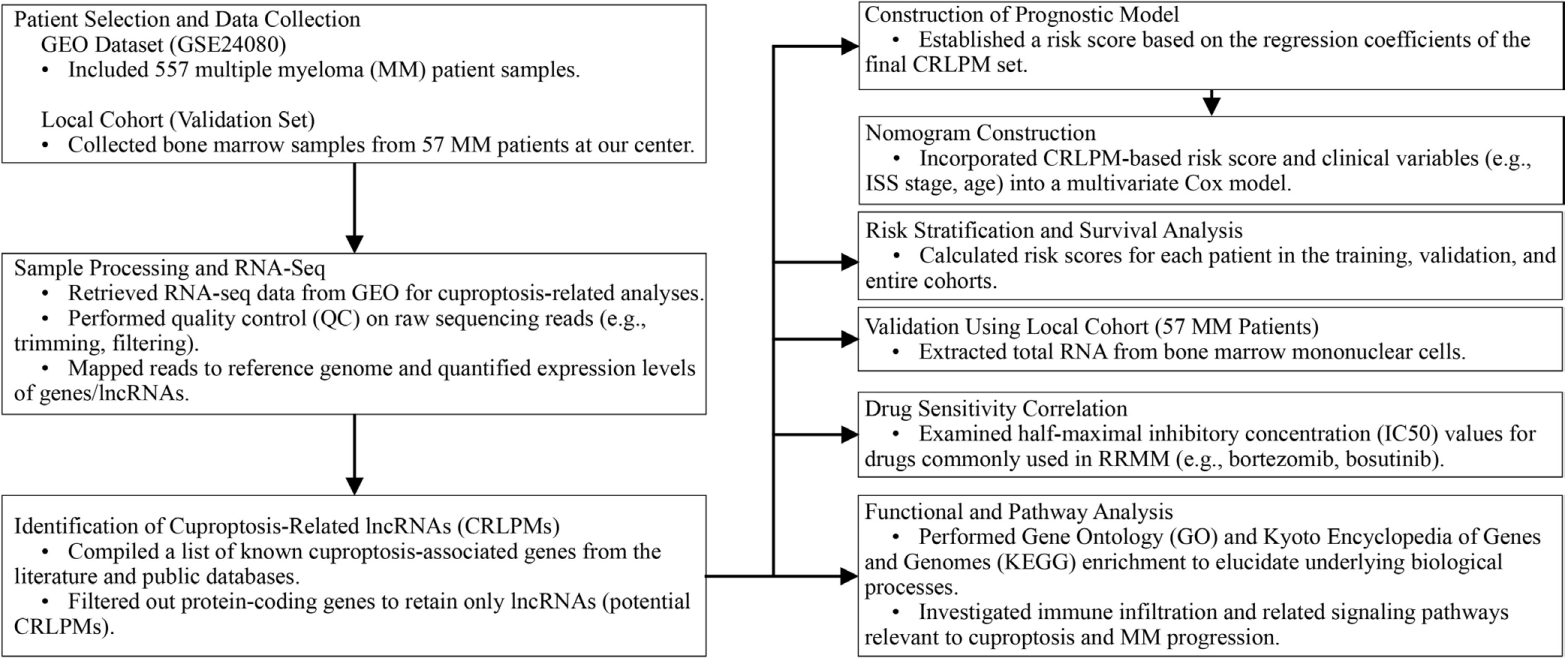
Displays a flowchart for the study.

Univariate Cox regression analysis was performed on CRLPMs to identify those significantly associated with OS. Significant lncRNAs were then subjected to LASSO‐Cox regression to avoid overfitting and select the most relevant ones. The final lncRNAs were used to construct a risk score model, calculated by multiplying each lncRNA's regression coefficient by its normalized expression level. Functional enrichment analysis was conducted using the “clusterProfiler” “Pheatmap.” Cox multivariable regression models established prognostic risk models, with internal validation via bootstrap resampling and calibration tests. Decision curve analysis evaluated the clinical benefit of the model, and survival outcomes were analyzed using log‐rank tests and KM curves. All analyses were performed using RStudio (Version 1.4.1717) and R (Version 4.2.2) [R Core Team, 2024]. The flowchart of the overall study is in Figure 1.

## 3 Results

### 3.1 Development and Validation of Prognostic Indicators for Cuproptosis‐Associated Long Noncoding RNAs

**FIGURE 2:**
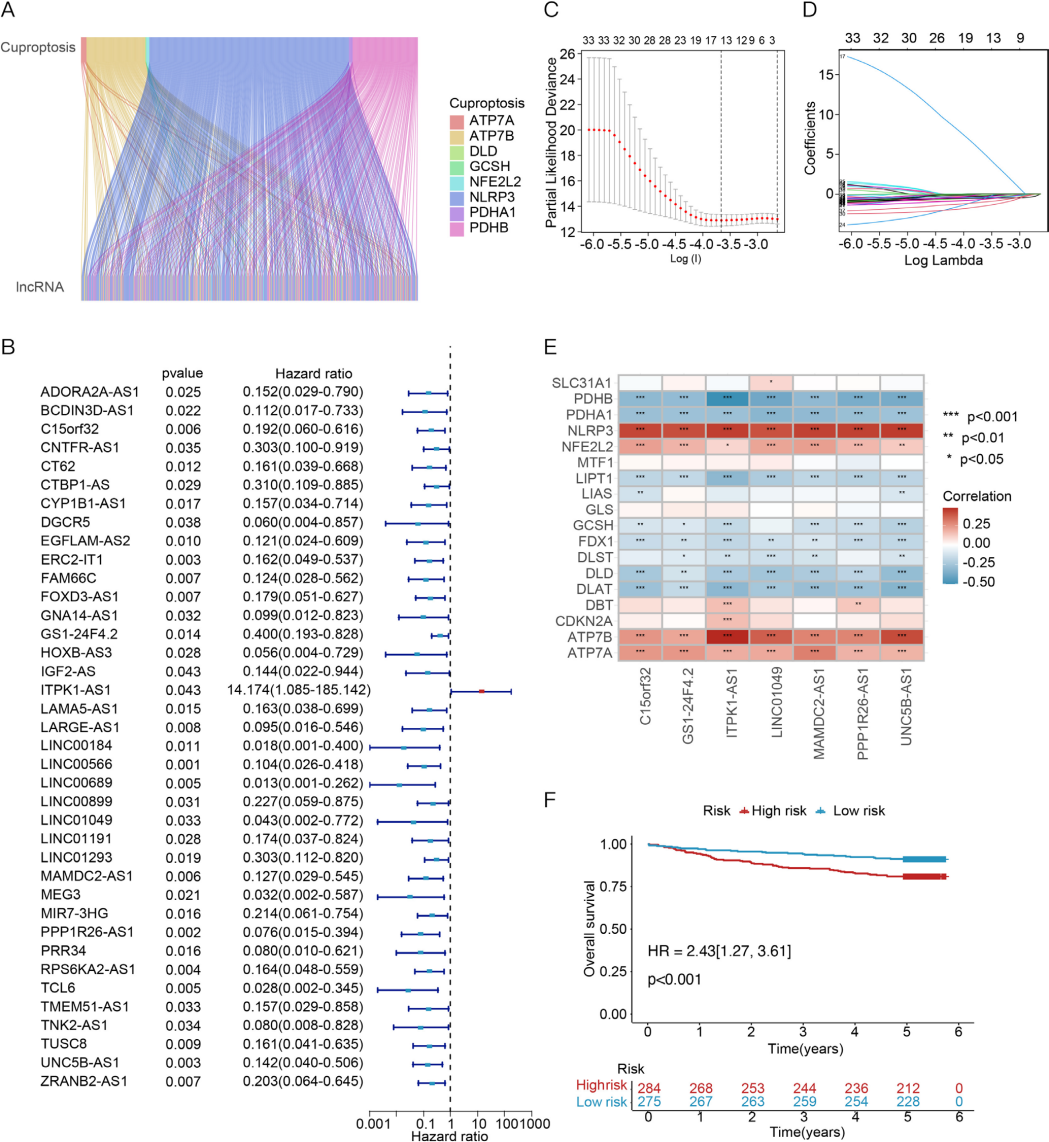
Screening and prognostic analysis of lncRNAs associated with cuproptosis. (A) The coexpression relationships between 19 cuproptosis‐related genes and 2137 cuproptosis‐associated lncRNAs were depicted using a Sankey diagram. (B) The prognostic significance of cuproptosis‐related lncRNAs was determined through univariate Cox regression analysis. (C, D) Lasso‐Cox regression analyses were performed to construct predictive models for prognosis. (E) Correlation analysis demonstrated the relationships between the 19 cuproptosis‐related genes and the 8 prognostic cuproptosis‐associated lncRNAs. (F) Kaplan–Meier survival analysis compared the high‐ and low‐risk groups in terms of OS. Statistical significance is indicated as *p < 0.05, **p < 0.01, and ***p < 0.001.

To explore the association among CRLPMs, protein‐coding genes were excluded from a dataset of 557 MM cases (GSE24080) in the GEO database. This process resulted in the identification of 19 cuproptosis‐related genes, while Pearson correlation analysis revealed 2137 cuproptosis‐associated lncRNAs. A Sankey plot (Figure 2A) was then used to visualize the links between these genes and corresponding lncRNAs.

In Figure 2B, the univariate Cox analysis results for 21 CRLPMs are illustrated. Following this, LASSO‐Cox regression was applied to refine the selection, identifying 12 lncRNAs with significant trajectory changes in regression coefficients and consistent cross‐validation results (Figure 2C,D). A final subset of eight survival‐associated CRLPMs was chosen through multiple stepwise Cox regression analysis to construct the risk score models.

**FIGURE 3:**
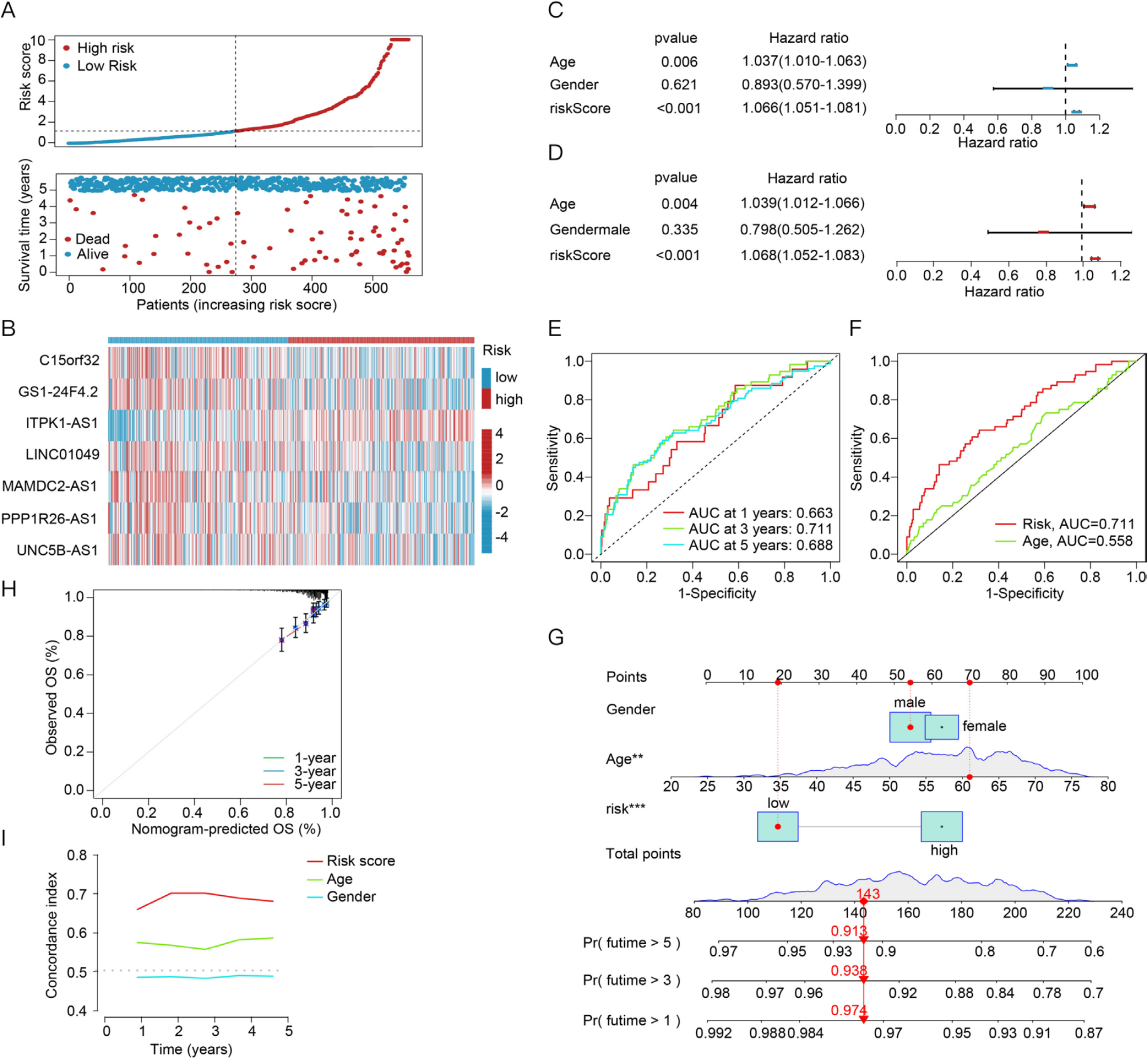
Illustrated the development and validation of a prognostic model for lncRNAs associated with cuproptosis in MM. (A) The distribution of risk scores and survival status among MM patients was shown. (B) A heatmap showcased the prognostic markers and OS. (C) Univariate analysis was performed to assess the prognosis based on the risk score. (D) Multivariable analysis was conducted to evaluate the prognosis based on the risk score. (E) The TimeROC curve predicted the 1‐, 3‐, and 5‐year OS for MM patients. (F) The C‐index demonstrated that the risk model exhibited better predictive accuracy compared to other clinical parameters. (G) A nomogram was constructed using the CRLPMs to predict OS. (H) Calibration curves were employed to predict the 1‐, 3‐, and 5‐year OS. (I) Decision curve analysis assessed the roles of the risk score, age, and gender in determining the prognosis of MM.

To explore the link between the selected CRLPMs and cuproptosis‐related genes, we generated a heatmap (Figure 2E). In the training cohort, individuals were stratified into high‐ and low‐risk groups based on the median risk score. KM analysis showed that high‐risk patients experienced markedly shorter overall OS compared to the low‐risk group (*p*‐value < 0.05). These significant survival discrepancies were mirrored in both the validation and entire cohorts (HR = 2.43 [1.27, 3.61], *p*‐values < 0.05; Figure 2F). Additionally, risk scores and patient survival statuses were mapped, and a heatmap illustrated the expression patterns of the 12 lncRNAs across the two risk categories (Figure 3A,B).

### 3.2 Evaluation of Cuproptosis‐Associated lncRNAs as Independent Prognostic Markers for Overall Survival

**FIGURE 4:**
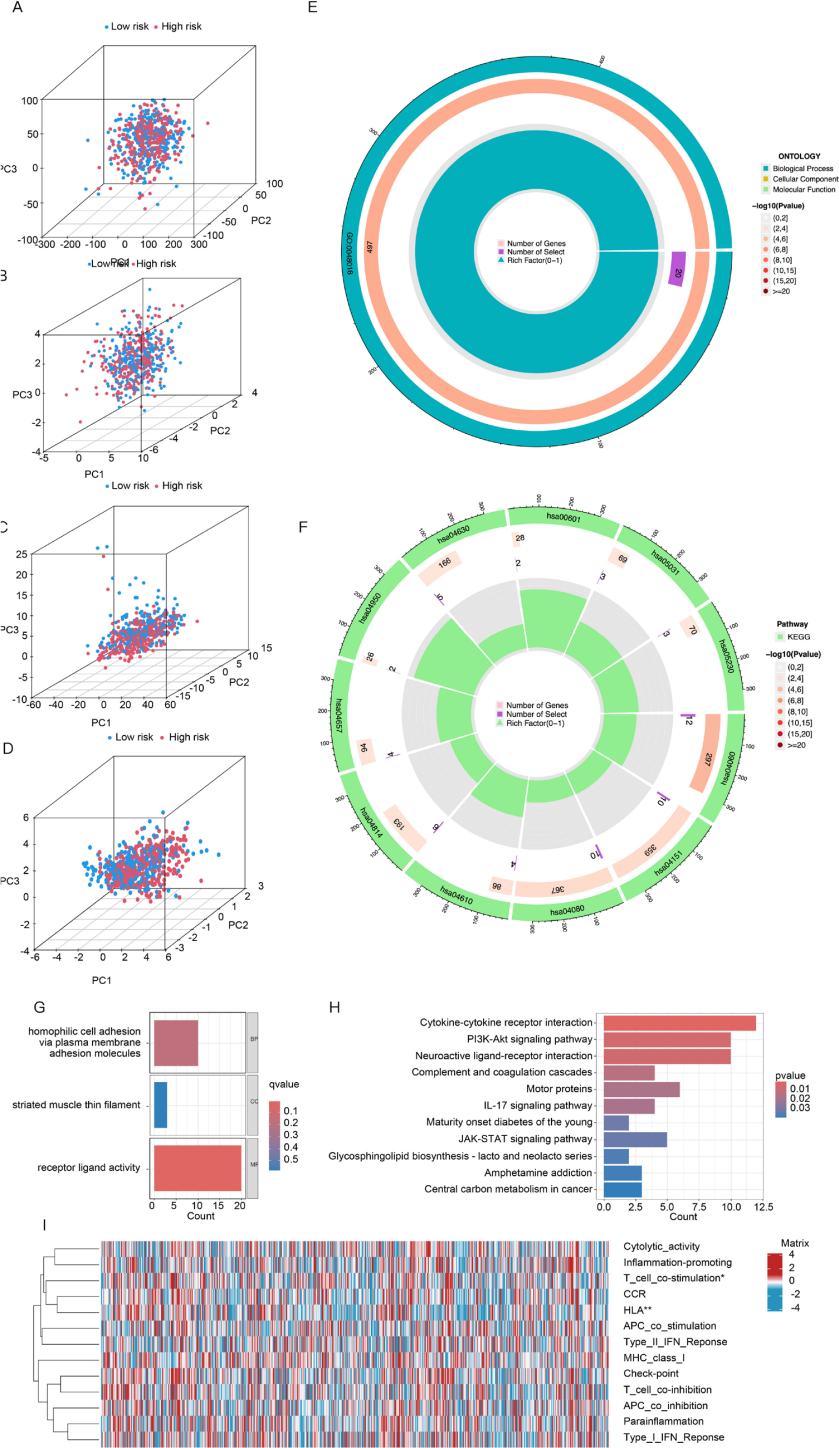
Presented the results of GO and KEGG pathway enrichment analysis. (A–D) PCA was used to compare the high‐ and low‐risk groups based on different gene sets, including all genes, cuproptosis‐related genes, and CRLPM prognostic markers. (E, F) The results of GO and KEGG pathway analyses were visualized using a circle diagram. (G) The top 10 enriched GO terms were illustrated in a barplot. (H) A barplot displayed the top 30 enriched KEGG terms. (I) The distribution of tumor‐infiltrating lymphocytes, based on single‐sample gene set enrichment analysis algorithms, was depicted in a heatmap for the high‐ and low‐risk groups of MM. Statistical significance was denoted as *p‐value < 0.05, **p‐value < 0.01, and ***p‐value < 0.001.

To appraise the predictive capability of the prognostic model, both univariate and multivariate Cox regression analyses were conducted. The univariate analysis revealed that age, gender, stage, and risk score were all statistically significant (Figure 3C,D). Receiver operating characteristic (ROC) curves further demonstrated the diagnostic utility of CRLPMs for OS, yielding area under the curve (AUC) values of 0.633 at 1 year, 0.711 at 3 years, and 0.688 at 5 years (Figure 3E). Both the C‐index and ROC findings underscored that this prognostic model outperformed other clinical metrics, such as age and risk, in accurately predicting patient outcomes (Figure 3F). To provide quantitative projections of clinical outcomes in MM, a prognostic nomogram integrating the risk score and clinical variables was generated (Figure 3G). Calibration plots demonstrated strong concordance between predicted and observed results (Figure 3H). The clinical significance of the CRLPMs was further investigated by examining their relationship with clinical features, revealing notable variations in risk scores across different disease stages (Figure 3I). Finally, principal component analysis (PCA) confirmed a distinct separation between high‐ and low‐risk groups, thus effectively stratifying MM patients based on CRLPMs (Figure 4A–D).

We identified 574 differentially expressed genes (DEGs) between the low‐ and high‐risk groups and performed functional enrichment analyses using the GO and KEGG databases to explore the molecular functions and pathways these DEGs may regulate. GO analysis revealed significant enrichment in biological processes like homophilic cell adhesion (ES = 10.0), striated muscle thin filament (ES = 2.6), and receptor ligand activity (ES = 20.0) (Figure 4E,G). KEGG analysis linked CRLPMs to key signaling pathways, including PI3K‐Akt (ES = 10.0), IL‐17 (ES = 2.6), and JAK–STAT (ES = 5.0) (Figure 4F,H). Additionally, differential analysis of immune function‐related factors showed that the high‐risk group had enhanced T cell co‐stimulation and HLA levels (Figure 3I). These findings provide valuable insights into the molecular mechanisms and potential biological functions of DEGs related to cuproptosis and its associated lncRNAs.

### 3.3 Validation of MM Samples and Evaluation of Drug Sensitivity and Personalized Treatment Potential of CRLPMs in RRMM

**FIGURE 5:**
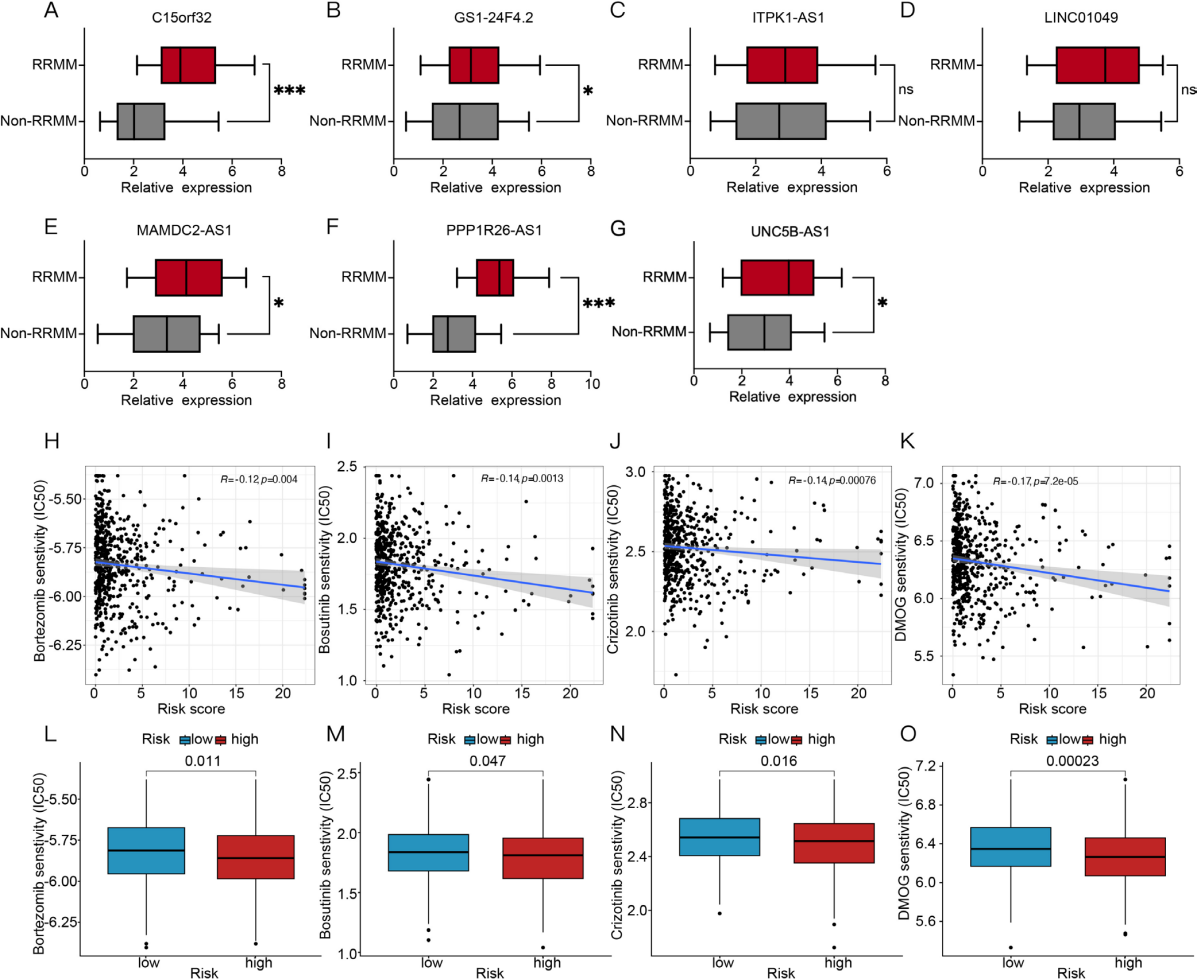
Validation of 57 MM patient samples and exploration of copper‐induced cell death targeted drugs in RRMM patients. C15orf32 (A), GS1‐24F4.2 (B), ITPK1‐AS1 (C), LINC01049 (D), MAMDC2‐AS1 (E), PPP1R26‐AS (F), UNC5B‐AS1 (G) levels of CRLPMs in RRMM and MM patients. Correlation analysis of drug IC50 and risk scores in Bortezomib (H), Bosutinib (I), Crizotinib (J), and DMOG (K). Drug sensitivity (IC50) of high‐ and low‐risk patients in RRMM for Bortezomib (L), Bosutinib (M), Crizotinib (N), and DMOG (O). These values (IC50) represent the half‐maximal inhibitory concentration.

BM samples were collected from 57 MM patients at our center. After extracting total RNA, qPCR was used to measure the expression changes of seven CRLPMs in both RRMM and non‐RRMM patients. The results showed that C15orf32 (Figure 5A), GS1‐24F4.2 (Figure 5B), MAMDC2‐AS1 (Figure 5E), UNC5B‐AS1 (Figure 5F), and PPP1R26‐AS (Figure 5G) were significantly increased in the RRMM group (p‐values < 0.05). However, there were no significant differences in ITPK1‐AS1 (Figure 5C) and LINC01049 (Figure 5D) levels between the groups. The lncRNA expression levels are determined via 2^‐ΔΔCt relative to a housekeeping gene (GAPDH).

To evaluate the feasibility of MAMDC2‐AS1‐based CRLPMs for personalizing RRMM therapy, we examined the correlation between patient risk scores and the half‐maximal inhibitory concentrations (IC50) of commonly administered RRMM drugs. Among 257 anticancer drugs, four—Bortezomib, Bosutinib, Crizotinib, and DMOG—showed significant differences in sensitivity between high‐risk and low‐risk groups (*p*‐values < 0.05). In our model, we used a standard 48‐h drug incubation period for viability and IC50 determination. Notably, these drugs had inferior IC50 values in the high‐risk group, indicating higher responsiveness, suggesting their potential therapeutic benefit for RRMM patients. Additionally, an inverse relationship between risk scores and IC50 values was observed for most RRMM drugs.

## 4 Discussion

In our study, we explored the relationship between CRLPMs in MM. Through correlation analysis, we included 2137 CRLPMs and refined this list to 12 lncRNAs using LASSO‐Cox regression. These lncRNAs were used to build a prognostic risk model, in which patients in the high‐risk group exhibited significantly shorter OS. Pathway enrichment analysis revealed significant associations between CRLPMs and known signaling pathways. The findings of this study contributed to our understanding of the role of CRLPMs in MM prognosis and underscore the potential of CRLPMs for personalized treatment strategies in RRMM.

Further analyses revealed meaningful differences in drug sensitivity for four of 257 anticancer agents—Bortezomib, Bosutinib, Crizotinib, and DMOG—implying potential therapeutic advantages for RRMM. Copper's redox properties, along with its ability to induce apoptosis and autophagy via mechanisms such as reactive oxygen species and proteasome inhibition [18], underscore its complex role in cancer biology [19]. Additionally, copper is vital for immune competence, influencing tumor immune evasion by modulating programmed cell death‐ligand 1 (PD‐L1) expression. Combining copper with specific compounds has shown enhanced antitumor efficacy [20, 21]. Studies have also suggested that copper can affect tumors via PD‐L1 expression [22]. Additionally, copper is crucial for maintaining immune competence, and its deficiency can impair immune function [23, 24], highlighting new opportunities for research on copper‐mediated cell death [25, 26]. In MM, the connection between copper metabolism and disease progression has been explored. Our investigation presented a novel prognostic model for RRMM by identifying seven cuproptosis‐associated lncRNAs, suggesting that these lncRNAs are significant predictors of OS in MM patients.

A notable discovery was that high levels of cuproptosis‐related lncRNAs correlated with aberrant activation of the PI3K‐Akt pathway [27], critical for cellular proliferation and survival [28]. Evidence suggests that inhibiting PI3K‐Akt can heighten Bortezomib sensitivity [29], possibly by reducing STMN1 expression [30]. Indeed, our data indicated that Bortezomib may be particularly effective in MM cells exhibiting elevated copper‐induced cell death, reinforcing the importance of this pathway in mediating drug resistance. Therefore, investigating the role of the PI3K‐Akt signaling pathway in Bortezomib resistance and cell proliferation could lead to the identification of novel targets for diagnosing and preventing the progression of MM. Our drug sensitivity tests also indicated that Bortezomib was particularly effective in MM cases with elevated copper‐induced cell death, which may be mediated through the PI3K‐Akt pathway. This dysregulated copper homeostasis triggers the accumulation of misfolded and damaged proteins within mitochondria, leading to the activation of the unfolded protein response (UPR) and other stress responses, which further exacerbate cellular damage.

We compared our data with those from previous studies specifically focused on RRMM. For instance, Rajkumar and Kumar [31] discussed the diagnosis and treatment of MM, including management strategies for RRMM patients, emphasizing the importance of gene expression profile changes in disease progression. Our study aligns with this emphasis but focuses on the specific expression of lncRNAs such as MAMDC2‐AS1 and its impact on the prognosis of MM. However, their study primarily concentrated on clinical treatment strategies and drug responses. Similarly, Kumar et al. [32] evaluated the prognosis of patients refractory to drugs, highlighting the potential role of biomarkers such as TP53 and *t*(4;14) in predicting outcomes. In their research, Kumar et al. highlighted the role of TP53 in predicting outcomes among patients with RRMM. Our study focused on cuproptosis‐associated lncRNAs, such as MAMDC2‐AS1, which are significantly upregulated in RRMM patients and linked to OS. These lncRNAs regulate cell death pathways and serve as potential biomarkers for the diagnosis, prognosis, and treatment of RRMM or MM. Our findings highlight the novel roles of these lncRNAs in disease progression and patient outcomes, addressing concerns raised in the peer review by providing a detailed comparison with established research on gene expression. While both studies address the natural course and treatment responses in RRMM, our research investigates the molecular mechanisms by analyzing lncRNA expression patterns. In summary, our research explores the prognostic value of specific cuproptosis‐related lncRNAs in RRMM patients by analyzing their expression patterns. While previous studies emphasize the significance of gene expression in RRMM, our study provides a focused analysis of specific lncRNAs, contributing to a deeper understanding of the molecular mechanisms and offering potential guidance for future research and therapeutic strategies.

Our findings on the upregulation of MAMDC2‐AS1 align with other studies identifying lncRNAs as significant prognostic markers in RRMM, and we observed similar correlations with poorer OS. However, our emphasis on MAMDC2‐AS1 as a particularly strong predictor, alongside the PI3K/Akt pathway's involvement, highlights novel aspects not extensively covered in earlier research. Possible reasons for these differences include variations in patient cohorts, methodological differences, and biological variability.

## 5 Conclusion

Our study identified a set of cuproptosis‐related lncRNAs that contributed to the overall risk score for MM patients. Among these, MAMDC2‐AS1 emerged as a key predictor of patient outcomes. By transitioning from a composite risk score to focusing on MAMDC2‐AS1, we aim to simplify and enhance the prognostic assessment for RRMM. MAMDC2‐AS1's strong association with patient prognosis suggests its potential as a standalone biomarker, offering a more straightforward and effective approach for predicting disease outcomes and guiding therapeutic decisions.

## Author Contributions

The data analysis for this study was conducted by Yifei Chen, who also drafted the manuscript. Database searching was performed by Yifei Chen. Data analysis was contributed by Jun Liu. The supervision and revision of the manuscript were carried out by Ying Zhu and Yifei Chen, who were involved in the entire study process.

## Ethics Statement

Informed consent was obtained from all patients or their legal guardians, and the study was conducted in accordance with the guidelines set by the Ethics Committee of Jiangdu People's Hospital and the Declaration of Helsinki.

## Consent

The patient, or the patient's legal guardian, hereby gives consent for the publication of the article titled “MAMDC2‐AS1 Induces Cuproptosis in Relapsed and Refractory Multiple Myeloma” in the journal Experimental and Therapeutic Medicine. The patient and/or guardian confirms that they have read the manuscript and understand its content. They acknowledge that the information contained in the manuscript may include details related to the patient's medical condition and treatment.

## Conflicts of Interest

The authors declare no conflicts of interest.

## Supporting information


**Table S1.** Primer sequences and qPCR conditions for each target.

## Data Availability

The data that support the findings of this study are available from the corresponding author upon reasonable request.
